# Patient preferences for growth hormone treatment in Japanese children

**DOI:** 10.1111/ped.14760

**Published:** 2021-08-25

**Authors:** Toshiaki Tanaka, Takahiro Sato, Akira Yuasa, Takeshi Akiyama, Adeeb Tawseef

**Affiliations:** ^1^ Tanaka Growth Clinic Tokyo Japan; ^2^ Medical Affairs, Rare Disease Pfizer Japan Inc Tokyo Japan; ^3^ Corporate Affairs, Health and Value Pfizer Japan Inc Tokyo Japan; ^4^ RWE & HEOR IQVIA Solutions Japan K.K Tokyo Japan

**Keywords:** children, conjoint analysis, discrete choice experiment, growth hormone deficiency, patient reported outcome

## Abstract

**Background:**

There are not clear evidence to date evaluating patients' and caregivers' preferences for the recombinant‐human growth hormone (r‐hGH) injection in children in Japan. This study aimed to quantitatively evaluated the factors driving preferences for daily r‐hGH injections among Japanese children with growth hormone deficiency (GHD) or their caregivers and to determine the relative importance of treatment delivery factors.

**Methods:**

This study was performed among Japanese children with GHD or their caregivers who visited a specialized clinic in Japan as part of their routine care. The participants were asked to complete a web‐based discrete choice experiment (DCE) questionnaire.

**Results:**

Choice‐based conjoint analysis was used to evaluate the relative importance of the attributes of the choice predictors and determine utility scores for each attribute. Of the 47 respondents who participated in this study, 41 were caregivers who responded on behalf of the patients, the remaining six were patients who completed the DCE themselves. The injection schedule was found to be the most important factor for both patients and caregivers; a once‐weekly injection schedule was preferred over a daily injection schedule. Storage and preparation was deemed more important to patients than it was to caregivers, with patients preferring storage at room temperature even if it required additional mixing (reconstitution). Both patients and caregivers showed a clear preference for devices that offered a dose‐setting memory.

**Conclusions:**

A less frequent injection schedule may enhance adherence to r‐hGH treatment and expected improve quality of life for GHD patients over the long term.

Recombinant human growth hormone (r‐hGH) has been an established therapy for growth hormone deficiency (GHD) in children and adults for more than three decades.^1^ Even before the introduction of r‐hGH, the use of pituitary‐extracted human GH was approved in Japan in 1975 for the treatment of GHD.^2^ In Japan, less than 1% of the pediatric population has been diagnosed with some form of GHD.^3^, ^4^ It is well known that r‐hGH treatment in Japanese children with GHD has a positive effect not only on growth promotion, but also on body composition and quality of life (QoL).^5^, ^6^

Growth hormone used to be administered daily by subcutaneous injection; however, even after long‐term GH treatment, near adult height remains unsatisfactory in Japan because the therapeutic dose used is lower than that used in the US and Europe.^3^, ^4^ Despite ongoing improvements in treatment, the burden of a daily subcutaneous injection regimen over several years remains inconvenient and painful, and this may affect adherence to treatment by patients and their caregiver's perspectives.^7^, ^8^ Adherence to treatment is crucial as it impacts clinical outcomes and QoL, and increases health expenditure.^9^, ^10^, ^11^ Therefore, to improve treatment outcomes, ameliorate injection fatigue, and increase adherence to treatment, a variety of long‐acting formulations of GH (LAGH) are currently in various phases of clinical study.^12^, ^13^ These treatment options maybe more applicable to patient populations who require additional care or monitoring, such as pediatric populations. Reducing injection frequency from once daily to once weekly or longer has the potential to improve patient adherence.^14^

An important deciding factor for patients and clinicians before initiating a treatment regimen is the frequency schedule of r‐hGH injections.^15^ Understanding patients' preferences for the r‐hGH injection regimen may improve patient satisfaction and adherence to treatment. Other treatment parameters, such as device attributes, safety, effectiveness, or mode of administration, as well as injection preferences, such as frequency or storage options, are factors that influence patient preferences for treatment.^15^ However, the frequency of preference for r‐hGH injection schedule in treating patients with GHD is not a clear concern, as currently only daily type is commercially available for treatment.^16^, ^17^, ^18^ Patient preference research allows identification of the preferences for various treatment options and potential trade‐offs that participants are willing to make.^19^ Patient preference for treatment is an essential dimension of health care that should be incorporated into decision making.

To our knowledge, to date there has been no study evaluating patient and caregiver preferences for a r‐hGH injection schedule nor injection device using a discrete choice experiment (DCE) in children with GHD in Japan. DCE is a quantitative method widely used in health care to elicit preferences from participants in the absence of revealed preference data. In this method, participants are presented with a series of hypothetical alternative scenarios containing several attributes, each of which may have several levels. Participants are asked to state their preferences between two or three competing scenarios.

The objective of this study was to evaluate quantitatively the factors driving preferences for r‐hGH injections among Japanese children with GHD or their caregivers and, additionally, to determine the relative importance of treatment delivery attributes, such as frequency of administration.

## Methods

This study was performed among Japanese children with GHD or their caregivers who visited a specialized clinic in Japan. The study was conducted in accordance with the Declaration of Helsinki and was approved by the Institutional Review Board (IRB) of the Specified Non‐profit organization Clinical Research Promotion Network Japan (1‐4‐9, Itachibori, Nishi‐ku, Osaka, Japan; No. CR‐IRB‐0115) and the non‐profit organization MINS (5‐20‐9‐401 Mita, Minato‐ku, Tokyo, Japan; No. MINS‐REC‐200218).

### Study design

This study was a cross‐sectional study, utilizing online tablet devices with support staff from the Site Management Organization (SMO). Clinician‐diagnosed pediatric patients (aged 3–17 years) and their caregivers were recruited between June and July 2020. The participants completed a DCE via an online questionnaire to determine their preferences for the r‐hGH injection regimen and injection device features. Product profiles for this study comprised features from both injection regimens and device attributes, each with two levels, and the choice tasks were presented to the participants based on the product profiles. Each participant was asked to complete 15 choice tasks, which were determined by experimental design. Each participant was presented with a different set of profiles to preclude the occurrence of association by chance. The number of responses was determined within the range expected to be acceptable to the respondents. The survey screen was also reviewed by practicing GHD clinicians, who examined the survey for understandability, comprehension, and layman terminology so that the survey could be easily understood by the participants. This study was validated by including a section at the end of the questionnaire to check the participants' understanding of the questions (Appendix S1).

Qualitative or quantitative approaches may be used to evaluate patient preference data, according to the US Food and Drug Administration (FDA) guidelines for patient preference information.^20^, ^21^ Qualitative methods to study patient preferences include group, individual, and individual/group methods, whereas quantitative approaches include discrete choice‐based, ranking, indifference, and rating methods.^20^

### Inclusion and exclusion criteria

Patients, or their respective caregivers, who met all the inclusion criteria were enrolled for the study. Inclusion criteria included age 3 years or older and less than 18 years on the day of screening. Patients on daily r‐hGH injections for GHD for at least 6 months prior to the day of screening were included in the study. Patients or their caregivers with the ability to understand the Japanese web questionnaire and not affected by cognitive limitations, as assessed by the clinical staff on site (i.e., the principal investigator), were included. Patients were also required to provide evidence of a personally signed and dated informed consent document and/or an assent form (for applicable ages), indicating that the patient (or a legally acceptable representative) had been informed of all pertinent aspects of the study. Patients who at the time of the study, were enrolled in a clinical trial or any other investigative study and who were treated with r‐hGH injections for non‐GHD related indications, as determined by the investigator, were excluded from the study.

### Sample size

The sample size was calculated based on the current prevalence of GHD among the pediatric population in Japan (currently under 1% nationally) and the available literature on sampling rare disease populations in Japan.^3^, ^22^ Specifically, when conducting a DCE, the sample size is calculated based on the number of choice tasks (T), the number of alternatives (A), and the number of analysis cells (C), which is equal to the largest number of levels for any of the attributes. As a rule of thumb, this study used the sampling size equation suggested by Johnson and Orme to calculate the sample size.^23^, ^24^ Therefore, a sample size of 47 respondents was calculated to be enough to draw statistically significant conclusions. In the end, this study was able to collect 47 respondents.

Furthermore, as with other DCE studies within GHD, such as one conducted in the US population,^25^ the study believed that the sample size reflected the pediatric population in Japan and would yield the desired statistical significance so that relevant conclusions could be drawn.^26^, ^27^ Participants were selected from among the patient population who visited the principal investigators' clinic as per their routine clinical care. All the participants and their respective caregivers were known patients of the principal investigator.

### Discrete choice experiment

The DCE choice tasks that each participant completed were determined via an experimental design based on predefined attributes and the grid levels. The DCE choice tasks consist of pictures and text and were designed for an intellectual level of 7 years of age and older; responses from patients under 7 years of age were assumed to be proxy responses (mainly parents). Eligible participants completed an online DCE questionnaire to indicate their preferred options from a choice of two across 15 tasks, The participants had to trade off the following five attributes: storage and preparation of injection medication, dose setting on the injection device, type of injection device, maintenance of injection device, and injection regimen (Table 1).

### Procedure

Interested patients and caregivers responded using an iPad tablet device, and consent and explanation documents were reviewed on the tablet screen. Participants enrolled in the study were asked to complete the DCE questionnaire, a series of clinical outcome assessments, and a brief demographic questionnaire. All the data, including participants' consent, were captured electronically using a web‐based interface.

The methodology used in this study is similar to that used by McNamara *et al*.^25^ Briefly, participants were given 15 choice tasks from which to elicit preferences regarding features of r‐hGH injections and injection devices (Table 1). Each choice task consisted of a different combination of device and injection features from which each participant was asked to choose. Through the DCE, participants' preferences for different devices and injection schedules and what they valued most that might influence their willingness to switch from their current r‐hGH device and schedule, were determined.

### Statistical methods

The primary and other analysis outputs (utility coefficients) were conducted using the SAP Qualtrics software (Seattle, Washington and Provo, Utah in the United States; www.qualtrics.com; version 102020 of Qualtrics. Copyright © 2019 Qualtrics). Choice‐based conjoint analysis (part‐worths and Bayesian hierarchical modeling) was used to evaluate the relative importance of attributes as choice predictors and determine utilities for each attribute. Participant preferences were estimated at the individual level. Bayesian hierarchical modeling was used to estimate utilities for each attribute level, from which the relative importance of attributes as choice predictors could then be determined. Logistic coefficients were extracted at the individual respondent level for each of the attributes and levels from the grid of options used to construct the discrete choice task profiles.

Attribute utilities were estimated using part‐worth modeling. Utilities were derived from the task design information and the 15 task choices completed by each respondent using Bayesian hierarchical modeling. Feature importance is defined as the measurement of influence a feature (attribute) has when the respondent is choosing their preferred bundle. The higher the score, the more weight it carries in the decision‐making process. The average level utility is defined as the average calculation across respondents' individual utility scores. Table 1 shows the levels for each feature and is helpful in determining how significant a level is in contributing to a feature's overall importance. The optimal package, which maximizes customer/buyer preference and utility, is defined as the most preferred package across respondents.

## Results

### Demographics and baseline characteristics

A total of 47 participants (41 caregivers and six patients) provided complete responses to all questions and were included in the study (Table 2). Patients receiving treatment ranged from 4 to 17 years old, and the mean age was 11.1.

### Respondent preferences

**Fig. 1:**
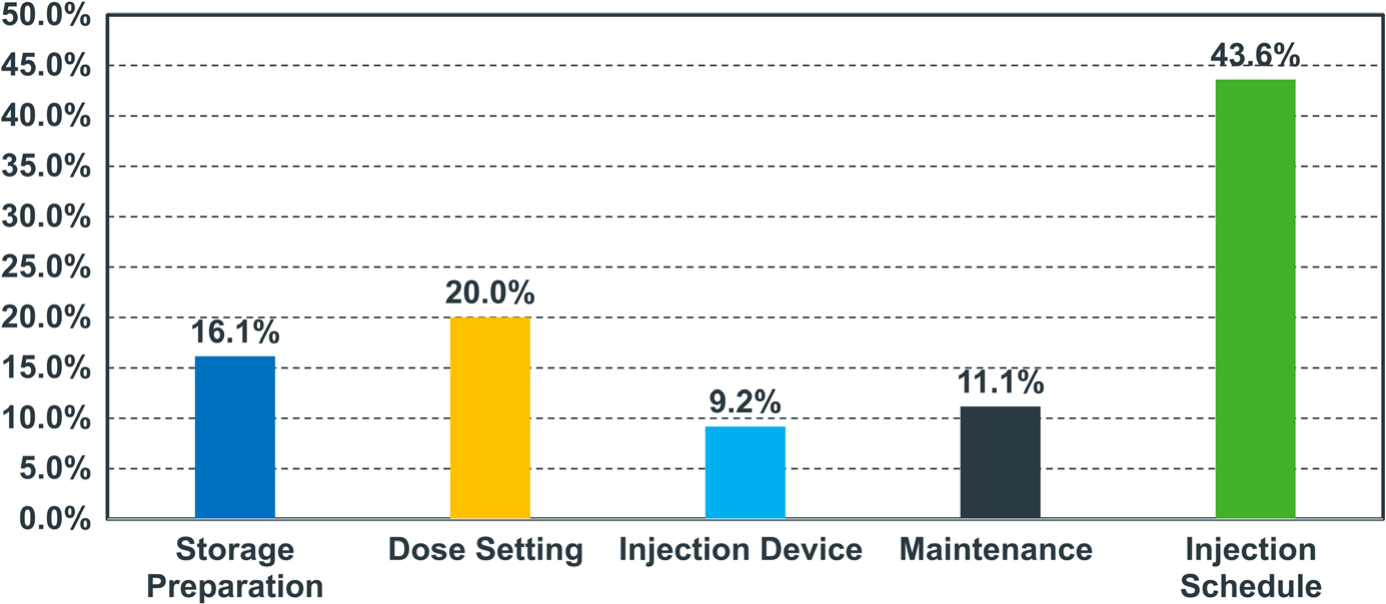
Relative importance from all responses (*N* = 47).

The most important feature in the treatment scenario was the injection scheduling, where 43.6% of respondents preferred injection scheduling as a desirable feature (shown in Fig. 1).

### Average conjoint utility scores

**Fig. 2:**
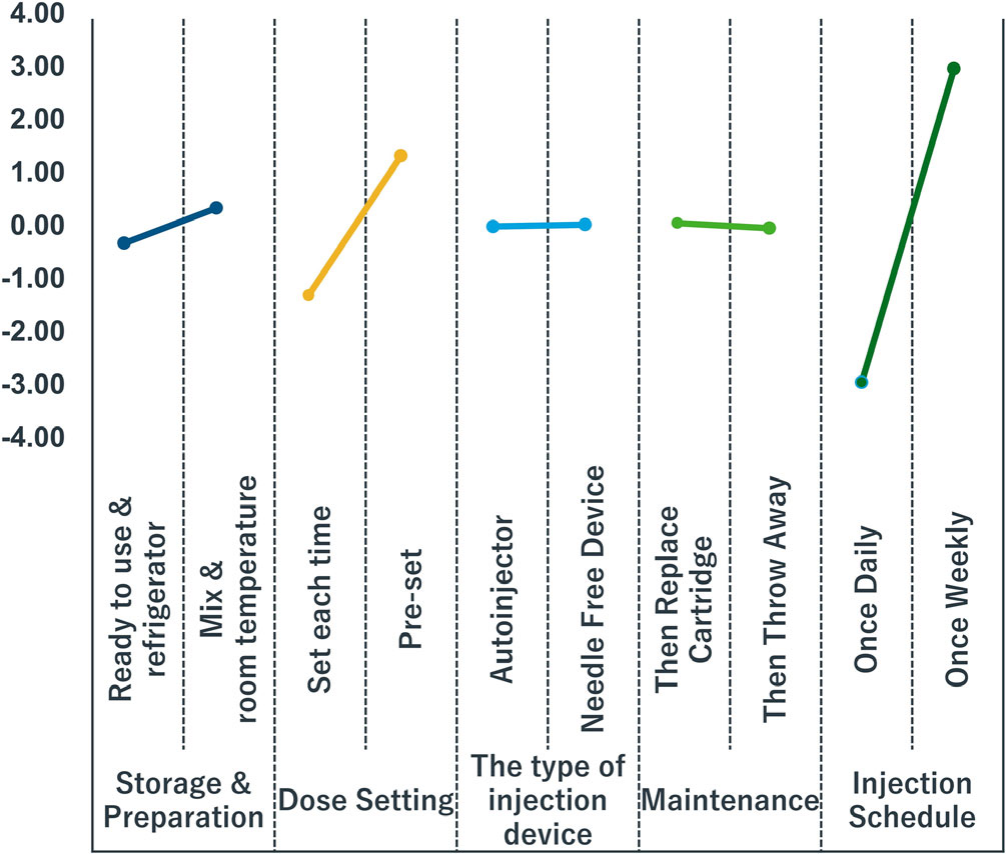
Average conjoint utility scores of selected attributes from all responses (*N* = 47).

The storage and preparation attributes showed neutral results. Overall, there was a slightly higher preference for 'ready to use and store in a refrigerator' than for 'mix (reconstitution) and store at room temperature' (Fig. 2). There was a clear preference for devices with features to set the dose the first time, and then the device remembers the setting for the remaining injection schedule. The preference for the type of injection device was neutral between needle‐free devices and autoinjector (pen) devices. Overall, the preference for device maintenance (replace cartridge/reusable pen devices vs disposable pen devices) was neutral with an average utility part‐worth value of 0.05 (for all respondents); however, patients had a stronger preference (an average utility part‐worth value of 1.71) for using pens that could be used by replacing cartridges, while caregivers had 0.20 part‐worth preference for disposable pen devices (Appendix S2). The preference for a once‐weekly injection schedule was strongly favored over once daily, with an average utility for the once weekly injection schedule of a part‐worth of 3.0 for all responses (Fig. 2); an average utility for the once‐weekly injection schedule of a 3.4 part‐worth for patients and a 2.9 part‐worth for caregivers (Appendix S2).

### Optimal package

Table 3 shows the optimal packages calculated from the analysis for both the patient and parent/caregiver response groups. While dose setting (set and remember), injection device (needle‐free) and injection schedule (once weekly) preferences were the same for both groups, storage preparation and maintenance preferences were group specific.

## Discussion

This study explored the preferences among Japanese children with GHD or their caregivers for r‐hGH injection frequencies and injection devices using the DCE method. Overall, patients and their caregivers showed a preference for a once‐weekly injection schedule over a once‐daily injection schedule compared to the other attributes considered in this study, such as storage and preparation, dose setting, injection device, and maintenance of injection device.

The daily injection burden of r‐hGH treatment and long treatment duration can be burdensome, affecting adherence to treatment.^28^, ^29^, ^30^ In addition, in a study of Japanese children and their families, it was reported that 70% of the subjects were burdened by the r‐hGH injection.^31^ In children, adherence to GH treatment is as important factor because poor adherence may affect clinical outcomes.^9^, ^10^, ^11^ Several efforts have been made to improve treatment adherence, including a non‐daily dosing regimen (every other day or three times weekly dosing),^32^, ^33^, ^34^, ^35^ use of injection pens or needle‐free devices, and minimization of medication reconstitution and storage requirements.^36^ However, adherence is difficult to monitor accurately, and only a few studies have shown that understanding of patient preferences for GH treatment is associated with increased adherence to treatment and improved clinical outcomes.^25^, ^37^ Therefore, there is a need to understand patients' preferences for GH treatment through reliable and robust approaches to enhance treatment outcomes. The DCE is a robust technique to elucidate quantitatively individual preferences over hypothetical scenarios.^19^ Such studies enable researchers to consider trade‐offs among attributes and help in tailoring interventions.^38^

A literature review revealed the paucity of data relating to patient preferences for r‐hGH injections and injection devices using DCE methods. Only one study to date conducted by McNamara *et al*. explored the trade‐offs and preferences of GHD patients or their caregivers, recruited from clinics in the US.^25^ The findings of that study showed that a less frequent injection schedule is the most preferred attribute for children with GHD, which is in line with our study. Daily injections cause inconvenience and discomfort for patients, whereas a more manageable weekly injection schedule has the advantage of patients having to spend less time and experience fewer injections. Also, weekly injections may reduce the burden of treatment and cause less interference with daily life.^39^ On the other hand, a study conducted by Amereller *et al*. showed that adult patients treated with LAGH expressed fear of forgetting weekly injections compared to daily injections.^40^

A study conducted by Meinhardt *et al*. showed that there was highest preference for device features associated with ease of use, such as no mixing required and being stored at room temperature to avoid storage issues while away from home or traveling.^41^ In our study, patients showed a slightly higher preference for ready to use and storage in the refrigerator. However, it is important to briefly discuss why there were differences between the patients and caregivers over preferences for storage and preparation compared with the injection scheduling regimen. It could be that in Japan, and especially for pediatric and adolescent patient groups, the prime person responsible for administering the medications is the patient's caregiver and not the patients themselves. Therefore, the caregiver could prioritize what they most prefer over what the patient may prefer or find useful. Similarly, when we interpret the results from the perspectives of the patients, we notice that their preference for the storage and preparation option (over scheduling injection) is due to what they think will make their lives easier, as the injection scheduling is not relevant to them. One explanation as to why patients may not put as much preference to injection scheduling could be due to the nature of how they are reminded to take medication – most likely from their caregivers or parents. Therefore, patients may prefer a certain attribute, which is easier for them to comprehend and more relevant to their lives. However, the sample size for the patients (*n* = 6) is not statistically significant enough to draw meaningful conclusions. Patient needs and preferences are moving towards reducing injection pain.^7^ The results were the same for injection device in this study because people tend to prefer needle‐free options.

All recruited participants were enrolled exclusively from one specialized clinic in Japan, even though treatment for GHD is standard across different countries and regions. Racial and cultural bias should be minimal, as in this scenario only patient preferences from Japan contributed to the results. Therefore, one strength of the recruited sample evaluated is that the preferences were optimized for Japan.

The study used a sample size that was enough to meet the study objectives. However, there are certain limitations which need to be addressed. DCE studies are affected by hypothetical bias as they ask patients to evaluate hypothetical choices.^15^ Therefore, if confronted with a real choice, preferences may differ from our DCE results. In our study, children with GHD had only experienced a daily injection schedule, and they did not have experience or knowledge of hypothetical alternative injection schedules. The bias should be assessed because several factors may affect adherence, including self‐injection, age, socioeconomic status, choice of injection device, and duration of treatment. However, we could not assess these factors since we also considered both limitations of data collection and conduct feasibility in this survey.

The study was not pretested as the ease of understanding the questions was confirmed by clinical physician review. Validation of responses (Appendix S1) indicated that the patients generally understood their responses to the questions. Further, while our study determined attributes by expert review and pharmaceutical insight, the literature review was limited.

While this is only a single‐center oriented study, the site has the largest number of patients in Tokyo. There is a room for further large‐scale, multicenter research in the future. Even though this study has highlighted the importance of injection frequency we could not verify whether the preference for injection frequency can really lead to improvement in patients’ compliance/adherence. Studies are needed to evaluate adherence to weekly GH treatment, once it becomes commercially available.

Many decisions in health care take into consideration patients' choices for treatment options when treatments are burdensome, limited, or affect the patient's QoL. Research has shown that clinicians, as well as patients, often vary considerably in evaluating treatment options, and clinicians alone are not always able to predict their patients' preferences for any treatment or clinical outcomes.^42^, ^43^ Therefore, there is a need to have shared decision making in preference‐sensitive decisions, to improve adherence to the treatment.^44^

### Conclusion

The results of this study showed that patients prefer a once‐weekly injection schedule over a daily injection schedule. A less frequent injection schedule may possibly enhance adherence and compliance to r‐hGH treatment in the long term and will also improve QoL in children with GHD. The benefits of a less frequent injection schedule can be further explored using real‐world studies.

## Disclosure

Pfizer Japan Inc. fully funded the study and was involved in study design. Fees were paid to IQVIA Solutions Japan K.K. for study conduct, analysis, and reporting. The authors declare the following potential conflicts of interest with respect to the research, authorship, and/or publication of this article: TT has received an advisory fee and honoraria from Pfizer Japan Inc. However, he did not receive any payment regarding the development of this manuscript. TS and AY are full‐time employees of Pfizer Japan Inc. TA and AT are full‐time employees of IQVIA Solutions Japan K.K.

## Author contributions

TS and AY contributed to the conception and design of this study; TA and AT performed the statistical analysis and drafted the manuscript; TT critically reviewed the manuscript and supervised the whole study process. All authors read and approved the final manuscript.

**Table 1:** Attributes and levels grid

Attributes	Level 1	Level 2
Storage and Preparation	Ready to use and store in refrigerator	Need mixing (reconstitution) and store at room temperature
Dose setting	Set the dose each time	Set the dose the first time
Injection device	Autoinjector	Needle‐free device
Maintenance	Replace cartridge (Reusable)	Throw away (Disposable)
Injection schedule	Once daily	Once weekly

**Table 2:** Background demographics of all patients receiving treatment (*N* = 47)

	*N*	%
Patients’ age		
Age (mean)	11.1	
<7	4	8.5
7–12	25	53.2
13–17	17	36.2
Answer rejected	1	2.1
Gender		
Female	15	31.9
Male	32	68.1
Current device		
Disposable pen‐shaped devices	3	6.4
Reusable pen‐shaped devices	25	53.2
Automatic injection motorized device	11	23.4
Needle‐free device	8	17.0

**Table 3:** Optimal package

	Patients, response	Parents/Caregivers’ response
Storage and Preparation	Ready to use (no mixing) and must be stored in the refrigerator	Mixing the medicine before use and store at room temperature
Dose Setting	Set the dose the first time, then device remembers it	Set the dose the first time, then device remembers it
Injection device	Needle free device (pushes medicine through the skin using high pressure)	Needle free device (pushes medicine through the skin using high pressure)
Maintenance	Multi‐use, reusable pen with replacement cartridge	Multi‐use, disposable pen
Injection schedule	Once weekly	Once weekly

## Supporting information


**Appendix S1.** Validation of responses.

**Appendix S2.** Patient and caregiver responses.
