# Supplementary material for: Patient preferences for growth hormone treatment in Japanese children
Source: Pediatr Int. 2021 Aug 25;63(10):1185–91. doi: 10.1111/ped.14760 (PMC8596999; doi:10.1111/ped.14760)
Supplement: Supplementary file 1 — Appendix S1. Validation of responses. [file PED-63-1185-s002.docx]

Supporting Information 1: Validation of responses

| How easy or difficult did you find choosing between scenarios in the previous section? | | | |
| --- | --- | --- | --- |
| Very easy | | 6 | 12.8% |
| Somewhat easy | | 14 | 29.8% |
| Neither difficult nor easy | | 19 | 40.4% |
| Somewhat difficult | | 6 | 12.8% |
| Very difficult | | 2 | 4.3% |
| How well did you understand the scenarios in the previous section? | | | |
| Fully understood the scenarios | | 12 | 25.5% |
| Somewhat understood the scenarios | | 34 | 72.3% |
| Did not understand the scenarios | | 1 | 2.1% |
| To what extent did you understand the characteristics described within the scenarios in the previous section? | | | |
| Fully understood the characteristics | 8 | | 17.0% |
| Somewhat understood the characteristics | 38 | | 80.9% |
| Did not understand the characteristics (Breakdown: preparation of injectable drugs, type of injector and injection schedule) | 1 | | 2.1% |
