# Supplementary material for: Patient preferences for growth hormone treatment in Japanese children
Source: Pediatr Int. 2021 Aug 25;63(10):1185–91. doi: 10.1111/ped.14760 (PMC8596999; doi:10.1111/ped.14760)
Supplement: Supplementary file 2 — Appendix S2. Patient and caregiver responses. [file PED-63-1185-s001.docx]

Supporting Information 2: Patient and caregiver responses

|  | | Patient responses  N=6 | Caregiver responses  N=41 |
| --- | --- | --- | --- |
| Feature Importance | | | |
| Storage & Preparation | | 33.0% | 13.6% |
| Dose Setting | | 11.4% | 21.3% |
| Injection Device | | 22.9% | 7.1% |
| Maintenance | | 13.8% | 10.8% |
| Injection Schedule | | 18.9% | 47.2% |
| Average conjoint utility scores from selected attributes | | |  |
| Storage & Preparation | Ready to use & store refrigerator | -4.0 | 0.2 |
|  | Mix(Reconstitution) & store in room temperature | 4.0 | -0.2 |
| Dose Setting | Set the dose each time | -1.8 | -1.2 |
|  | Set the dose the first time | 1.8 | 1.2 |
| Injection Device | Autoinjector | 2.8 | -0.4 |
|  | Needle-free device | -2.8 | 0.4 |
| Maintenance | Then replace cartridge (Reusable) | 1.7 | -0.2 |
|  | Then throw away (Disposable) | -1.7 | 0.2 |
| Injection Schedule | Once-daily | -3.4 | -2.9 |
|  | Once-weekly | 3.4 | 2.9 |
